# Prognostic molecular biomarkers in chordomas: A systematic review and identification of clinically usable biomarker panels

**DOI:** 10.3389/fonc.2022.997506

**Published:** 2022-09-29

**Authors:** Franco Rubino, Christopher Alvarez-Breckenridge, Kadir Akdemir, Anthony P. Conley, Andrew J. Bishop, Wei-Lien Wang, Alexander J. Lazar, Laurence D. Rhines, Franco DeMonte, Shaan M. Raza

**Affiliations:** ^1^ Department of Neurosurgery, Division of surgery, The University of Texas MD Anderson Cancer Center, University of Texas, Houston, TX, United States; ^2^ Department of Sarcoma Medical Oncology, Division of Cancer Medicine, The University of Texas MD Anderson Cancer Center, University of Texas, Houston, TX, United States; ^3^ Department of Radiation Oncology, Division of Radiation Oncology, The University of Texas MD Anderson Cancer Center, University of Texas, Houston, TX, United States; ^4^ Department of Pathology, Division of Pathology-Lab Medicine Division, The University of Texas MD Anderson Cancer Center, University of Texas, Houston, TX, United States

**Keywords:** Chordoma, prognostic biomarkers, molecular, skull base, spine, systematic review, multivariate analysis

## Abstract

**Introduction and objective:**

Despite the improvements in management and treatment of chordomas over time, the risk of disease recurrence remains high. Consequently, there is a push to develop effective systemic therapeutics for newly diagnosed and recurrent disease. In order to tailor treatment for individual chordoma patients and develop effective surveillance strategies, suitable clinical biomarkers need to be identified. The objective of this study was to systematically review all prognostic biomarkers for chordomas reported to date in order to classify them according to localization, study design and statistical analysis.

**Methods:**

Using the Preferred Reporting Items for Systematic Reviews and Meta-Analyses (PRISMA) guidelines, we systematically reviewed published studies reporting biomarkers that correlated with clinical outcomes. We included time-to-event studies that evaluated biomarkers in skull base or spine chordomas. To be included in our review, the study must have analyzed the outcomes with univariate and/or multivariate methods (log-rank test or a Cox-regression model).

**Results:**

We included 68 studies, of which only 5 were prospective studies. Overall, 103 biomarkers were analyzed in 3183 patients. According to FDA classification, 85 were molecular biomarkers (82.5%) mainly located in nucleus and cytoplasm (48% and 27%, respectively). Thirty-four studies analyzed biomarkers with Cox-regression model. Within these studies, 32 biomarkers (31%) and 22 biomarkers (21%) were independent prognostic factors for PFS and OS, respectively.

**Conclusion:**

Our analysis identified a list of 13 biomarkers correlating with tumor control rates and survival. The future point will be gathering all these results to guide the clinical validation for a chordoma biomarker panel. Our identified biomarkers have strengths and weaknesses according to FDA’s guidelines, some are affordable, have a low-invasive collection method and can be easily measured in any health care setting (RDW and D-dimer), but others molecular biomarkers need specialized assay techniques (microRNAs, PD-1 pathway markers, CDKs and somatic chromosome deletions were more chordoma-specific). A focused list of biomarkers that correlate with local recurrence, metastatic spread and survival might be a cornerstone to determine the need of adjuvant therapies.

## Introduction

Chordomas are malignant bone tumors, of possible embryologic origin, arising anywhere along the neuroaxis (1), that are prone to both local recurrence and systemic relapse. Successful treatment of chordomas requires an experienced multidisciplinary team. The median overall survival of patients with chordoma is between 6.3 and 7.7 years (1, 2) with a 5-year overall survival around 70% in most series (2–4). For newly diagnosed patients, primary treatment consists of radical but safe resection; importantly, the extent of resection directly impacts outcomes in patients with skull base and spine chordomas. Over time, treatment of newly diagnosed chordomas has also evolved to incorporate adjuvant radiation, commonly with particle therapy (carbon-ion or proton-ion), particularly for skull base chordomas (5–8). While outcomes have improved over time, the rates of both local recurrence and distant metastases remain high. Ultimately, it is widely accepted that chordoma behaves across a biological spectrum – ranging from an indolent to a more aggressive phenotype with poorer survival and increased risk of local recurrence and metastatic disease. Unfortunately, there is still an unmet clinical need to develop effective systemic therapeutic agents that can be incorporated into the treatment paradigm for newly diagnosed and recurrent disease. To this end, clinical biomarkers that can be used to better tailor treatment and implement surveillance strategies for patients with chordoma are needed.

To meet this need for clinical biomarkers, several molecular studies have been conducted to identify therapeutic targets and prognostic markers in order to predict outcome (9–12). While many of the identified biomarkers have been validated in the research laboratory setting, the challenge lies in creating a biomarker panel that can be used in the clinical setting to guide real-time decision-making (13, 14). According to the US Food and Drug Administration (FDA), a biomarker is defined as a measurable characteristic that serves as an indicator of normal or pathological biological processes or responses to an exposure or intervention. Additionally, biomarkers can include molecular, histologic, radiographic or physiologic features (15). To be clinically utilized, there are certain critical features that any biomarker should include: 1) it should be non-invasive, easily measured, affordable, and produce rapid results; 2) it should be accessible from readily available sources, such as blood or urine; 3) it should have high sensitivity to allow early detection and have a high specificity in diseased samples; 4) the expression level of the biomarker should aid in risk stratification and possess prognostic value in terms of clinical outcomes; and 5) the biomarker should provide insight into the underlying disease mechanism (16). Specifically, a prognostic biomarker identifies the likelihood of a clinical event, disease recurrence, or progression in patients who have a medical condition of interest (17).

The objective of the present study was to review all prognostic biomarkers reported to date for chordomas in order to classify them according to localization, clinical utility, study design and statistical analysis.

## Methods

### Study design

**Figure 1:**
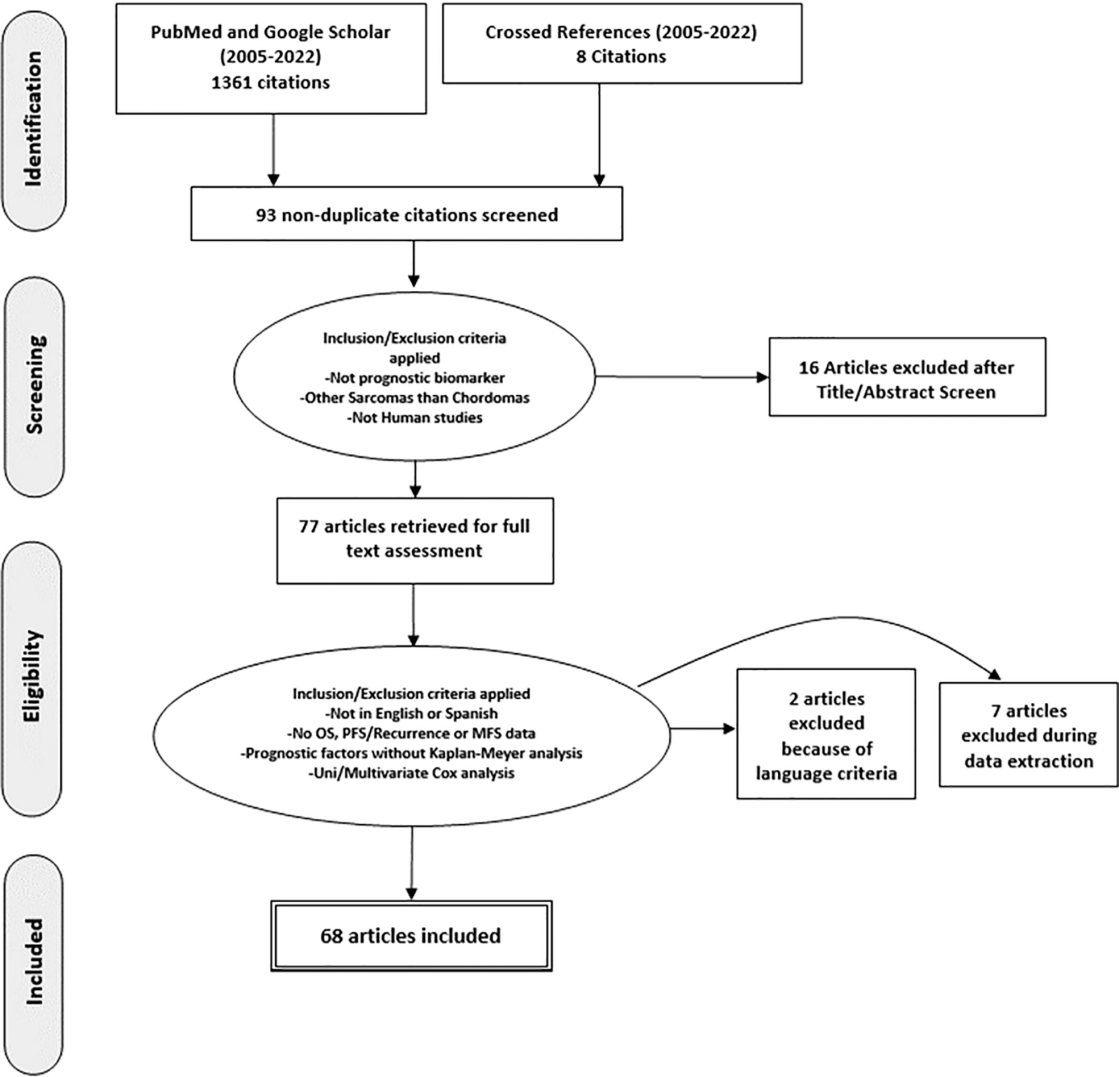
PRISMA-based flow chart of the study.

The current systematic review was performed according to the Preferred Reporting Items for Systematic Reviews and Meta-Analyses (PRISMA) guidelines (18). Studies were classified according to their design as *prospective* or *retrospective*. The level of evidence of each study was determined according to Oxford Centre for Evidence-Based Medicine (OCEBM) guidelines (19, 20). A systematic search was conducted in the PubMed/Medline and Google Scholar databases using the following terms: “chordoma”, “biomarker”, “overall survival”, “progression free survival”, “prognosis” and “biological marker”. Any discrepancies were resolved through consensus. The search strategy utilized is reported in **Figure 1**. Inclusion criteria were as follows: i) the study must have been a prospective clinical trial or a retrospective case-control/cross sectional study published in English with no start date restriction and until February 2022 (when analyses for this review were performed); ii) the studies had to have reported chordomas confirmed by pathology (either in the skull base, mobile spine or sacrum); iii) the studies must have been *time-to-event studies*, which determined Progression Free Survival (PFS), and/or Overall Survival (OS); iv) the outcomes must have been analyzed by employing Kaplan-Meier (K-M) analysis with log-rank test or a Cox proportional hazards analysis (Cox model); v) the study must have included a full biomarker description including the brand name of the assay used, the source/matrix, the measurement technique and the assay technology used; vi) studies must not have had overlapping patients.

Studies using other biomarker categories, such as susceptibility/risk, diagnostic, monitoring, predictive, pharmacodynamic/response or safety were excluded.

### Data abstraction

The extracted variables of each study include the following: Type of study, number of patients, localization of the primary tumor (skull base, mobile spine and/or sacrum), localization of the biomarker (cellular [nuclear, cytoplasm or cell membrane] or extracellular [blood or extracellular matrix]), assay technique for measurement, prognostic variable analyzed, non-parametric statistical approach, level of significance (p-value), and study design (prospective or retrospective). For classification purposes, we divide the biomarker as ‘positive’ or ‘negative’, according to its correlation with PFS or OS.

### Statistical analysis

The primary goal was to determine whether there was significant correlation between the biomarker and prognostic variable analyzed, either using Log-rank univariate analysis or Cox regression multivariate analysis. All statistical analyses were performed using Microsoft Excel 365 (Microsoft, Washington, USA) and GraphPad Prism 9.0 statistical software package (La Jolla, California, USA). Descriptive statistics was the main statistical technique. Categorical variables are presented as proportions and the continuous variables are presented with mean and standard deviation or median and interquartile range.

## Results

### Literature review

Sixty-eight studies met predetermined eligibility criteria and were included for data abstraction (**Figure 1**). Fourteen studies used the same cohort of patients with different biomarkers, so a total number of 1263 patients are duplicated (21–33). Two studies were multicenter, which includes different populations, increasing the strength of the study (21, 34). With regard to study design, 5 studies were prospective (7.3%) (26, 35–38) and 63 studies were retrospective (92.7%) (21–25, 27–34, 39–87).

### Identification of pertinent biomarkers

Data for a total of 3183 patients (not duplicated) were analyzed using different prognostic biomarkers. We excluded data from patients that was collected from the same hospital and in the same time frame. Of these, 3150 cases were adult patients (99%) and 33 were pediatric patients (1%); there were 1595 (50%) skull base chordomas, 1165 (36.6%) sacrococcygeal chordomas and 423 (13.4%) chordomas in mobile spine segments. A total of 103 biomarkers were analyzed in the 68 studies included in our analysis. Using the FDA classification system (15) we divided the biomarkers into 4 groups: molecular (82.5%; n=85), physiological (9.7%; n=10), histological (6.8%; n=7) and radiographic biomarkers (1%; n=1) (82).

Localization for each biomarker was different. A total of 40% were located in the nucleus (n=41), 22% in the cytoplasm (n=23), 14% in the cellular membrane (n=14), 7% in the extracellular matrix (n=7), and 16% from blood samples (n=17). The most common detection technique used was immunohistochemistry (IHC) in 66% of the studies (n=45) followed by polymerase chain reaction (PCR), serum protein analysis, and DNA sequencing in 20% (n=14), 9% (n=6) and 5% (n=3), respectively.

Three studies did not consider the expression of individual biomarkers, but instead they described a score using a group of biomarkers. One study describes an “miRNA-score”, which measures the nuclear concentration of different microRNAs (miR-1290; miR-574-3p; miR-1; miR-1237-3p; miR-155; miR-140-3p) using PCR-based techniques (42). Another study describes a “Systemic Inflammatory Index” (SII), which is obtained by multiplying blood platelet count and neutrophil count and dividing by lymphocyte blood counts (*P x N/L*) (41), and the last study describes an “Immunoscore” based on the expression of CD3+ and CD8+ cells in the tumor specimen, using IHC (58).

### Correlation of biomarkers with clinical outcomes

The clinical outcomes commonly evaluated in the selected studies were Progression free survival (PFS) and overall survival (OS). Only those biomarkers that demonstrated significant correlation with clinical outcomes in non-parametric tests were considered. Significant correlation of biomarkers with OS, PFS, or both, appeared in 35% (n=24), 23% (n=16) and 40% (n=27) of the studies, respectively. One study found that the lack of 1p36 LOH correlates with improved OS and PFS but did not report the HR and/or the CI (60). Because of this, we did not include their results in the following figures and analysis. Ultimately, 82 biomarkers were demonstrated to have statistical correlation in PFS and/or OS.

Elevated expression of 15 biomarkers was found to be associated with improved PFS and/or OS in chordoma patients (23, 24, 30, 31, 39, 55, 58, 62, 69, 76, 78, 84, 88). Among them, five biomarkers correlated with improved OS (elevated CD8+/Foxp3+ ratio, H-TERT promoter mutations, and high expression of SNF5 protein, c-MET receptor, and miRNA-1), eight biomarkers correlated with improved PFS (high *Immunoscore*, elevated PD1+ Tumor-Infiltrating Lymphocytes (TIL), high expression of PHLPP1 protein, Raf-1 and ERK proteins, elevated MMP-9/RECK protein ratio, and the high nuclear expression of miRNA-1290 and mi-RNA-1237-3p) and, 2 biomarkers were linked with both PFS and OS (elevated PD-L1+ TIL and high preoperative serum albumin level).

Increased expression of the remaining biomarkers (82%, n=67) correlated with poorer PFS and/or OS outcomes. Among them, the Ki-67 Level Index (LI) was the most frequent biomarker analyzed across the collection of studies (n=7, 10.3%). A high Ki-67 LI correlated with a shorter OS or PFS (even on COX regression analysis) (27, 33, 56).

Thirty-four studies analyzed the correlation between biomarkers and clinical outcomes using Cox multivariate regression and found that 35 biomarkers (43%) and 22 biomarkers (27%) were independent prognostic factors for PFS and OS, respectively. Although these biomarkers demonstrated a *p-value* less than 0.05, the confidence intervals (95% CI) varied greatly, partly due to limited sample sizes. To mention some examples, in one study, high *Brachyury* gene expression or increased number of alterations in chromosome 2p (CNA 2p) correlated with improved PFS in a sample of 37 patients, but the CI of both is wide and include the value 1 (45). Furthermore, other biomarkers like VEGFA and E-cadherin had a CI of almost 1 in a sample size of 151 patients (27).

**Figure 2:**
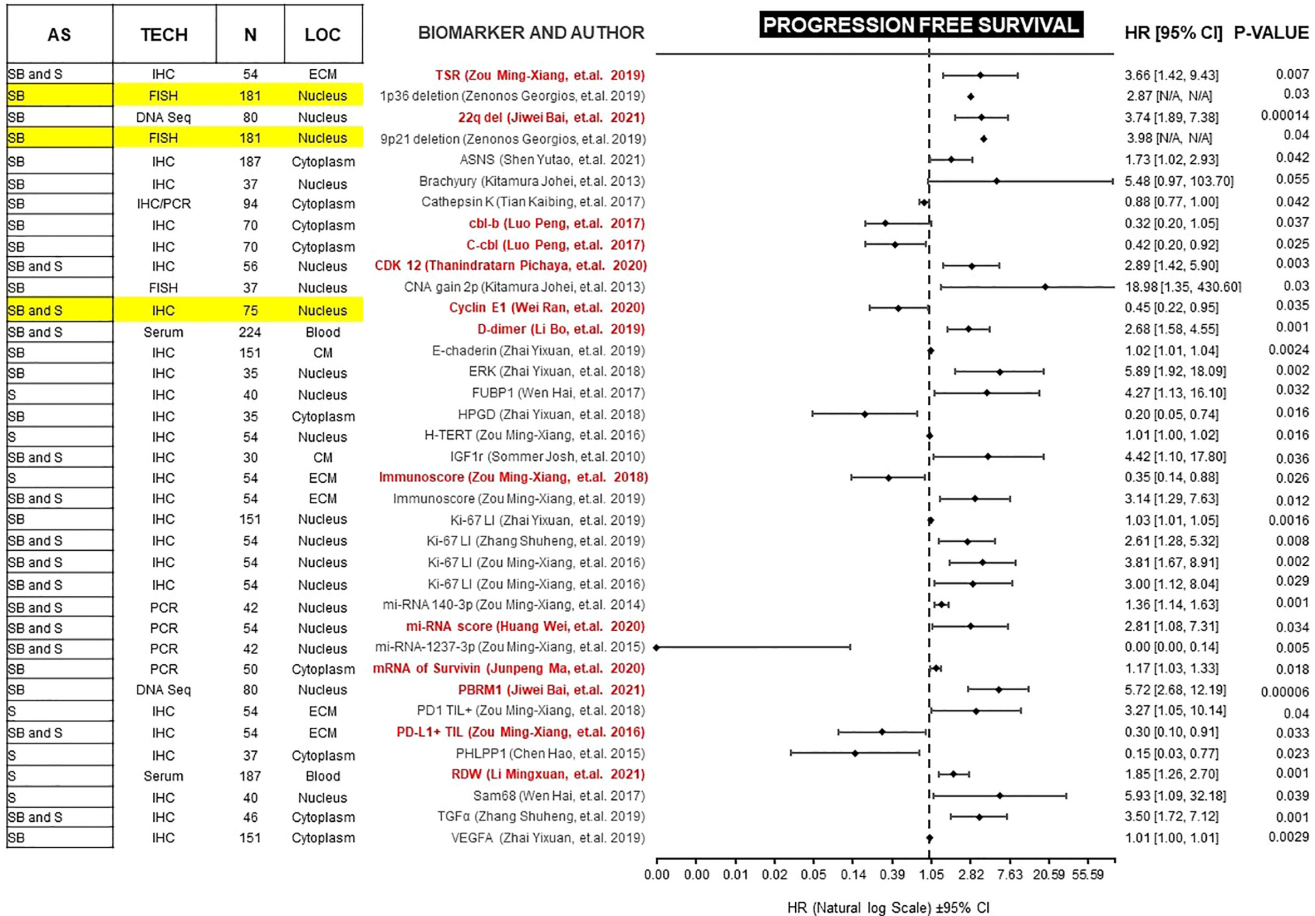
Forest plot showing biomarkers associated with PFS benefit on multivariate analysis. Those biomarkers with benefit in PFS and OS are marked in red. Prospective studies are marked in yellow. AS (Anatomic Site), TECH (Assay technique used), N (number of patients), LOC (Biomarker localization), SB (Skull base), S (Spine), IHC (Immunohistochemistry), PCR (Polymerase chain reaction), DNA Seq (DNA sequencing), FISH (fluorescence *in situ* hybridization), ECM (extracellular matrix), CM (Cell Membrane), TSR (Tissue-stroma ratio), PD-L1 TIL (Programmed death ligand 1, Tumor infiltrating lymphocytes), CDK (Cyclin-dependent kinase), TGFα (Tumor Growth Factor α), RDW (Red cell distribution width), CNA (copy number alterations), N/A (Not available).

**Figure 3:**
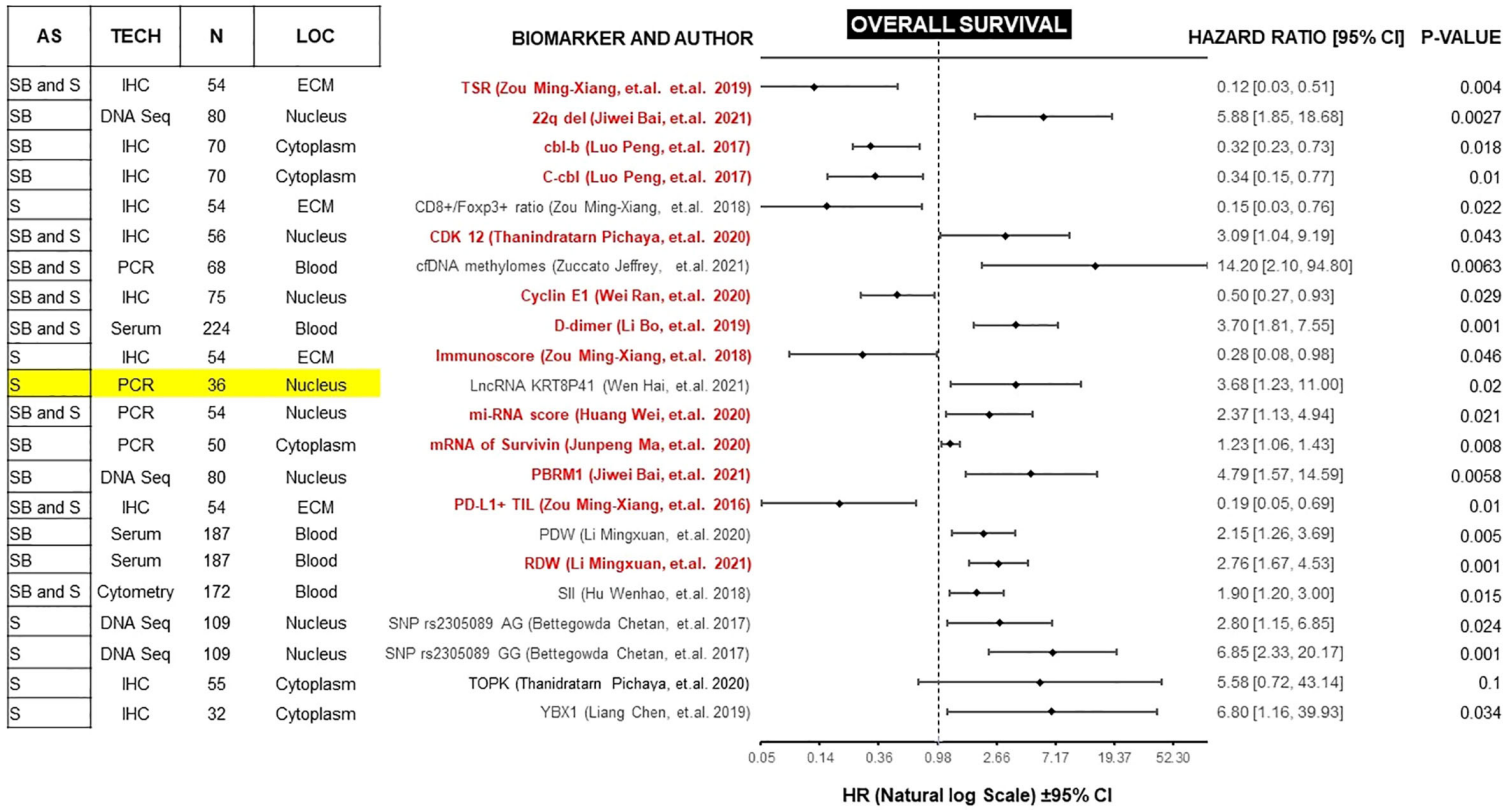
Forest plot showing biomarkers associated with OS benefit on multivariate analysis. Those biomarkers with benefit in PFS and OS are marked in red. Prospective studies are marked in yellow. AS (Anatomic Site), TECH (Assay technique used), N (number of patients), LOC (Biomarker localization), SB (Skull base), S (Spine), IHC (Immunohistochemistry), PCR (Polymerase chain reaction), DNA Seq (DNA sequencing), FISH (fluorescence *in situ* hybridization), ECM (extracellular matrix), CM (Cell Membrane), TSR (Tissue-stroma ratio), PD-L1 TIL (Programmed death ligand 1, Tumor infiltrating lymphocytes), RDW (Red cell distribution width), PDW (Platelet distribution width), LncRNA (Long non coding RNA).

Forest plots in **Figures 2** and **3**, help to clearly differentiate those studies with more statistical strength. Overall, 13 biomarkers (19%) had significant correlation with both clinical outcomes (**Table 1**).

**Table 1:** List of reported biomarkers with independent prognostic impact in PFS and OS (with Cox multivariate analysis).

Biomarker	Level of evidence	N	Localization	Assay Technique	HR os	OS 95% CI	p-value OS	HR PFS	PFS 95% CI	p-value PFS
22q del	IIb	80	Nucleus	DNA sequencing	5.88	1.85-18.68	***0.046***	3.74	1.89-7.38	***0.0024***
Cbl-B	IIb	70	Cytoplasm	IHC	0.32	0.234-0.734	***0.018***	0.32	0.20-1.054	***0.037***
C-Cbl	IIb	70	Cytoplasm	IHC	0.34	0.153-0.771	***0.01***	0.42	0.20-0.92	***0.025***
CDK-12	IIb	56	Nucleus	IHC	3.09	1.04-9.19	***0.043***	2.89	1.42-5.9	***0.003***
Ciclin E1	Ib	75	Nucleus	IHC	0.5	0.27-0.93	***0.029***	0.45	0.22-0.95	***0.035***
Immunoscore (Cd3+/Cd8+)	IIb	54	Extracellular matrix	IHC	0.28	0.08-0.98	***0.046***	0.35	0.14-0.88	***0.026***
mi-RNA Score	IIb	54	Nucleus	PCR	2.37	1.13-4.94	***0.021***	2.81	1.08-7.31	***0.034***
PBRM1	IIb	80	Nucleus	DNA sequencing	4.79	1.57-14.59	***0.006***	5.72	2.68-12.19	***0.00006***
PD-L1+ TIL	IIb	54	Extracelullar matrix	IHC	0.188	0.051-0.69	***0.01***	0.3	0.10-0.91	***0.033***
RDW	IIb	187	Blood sample	Hemocytometry	2.757	1.67-4.53	***0.001***	1.85	1.26-2.7	***0.001***
Survivin (mRNA)	IIb	50	Nucleus	PCR	1.23	1.06-1.43	***0.008***	1.17	1.03-1.33	***0.018***
Tissue-Stroma Ratio	IIb	54	Extracellular matrix	IHC	0.12	0.03-0.51	***0.004***	3.66	1.42-9.43	***0.007***
D-Dimer	IIb	224	Serum sample	Serum analysis	3.7	1.81-7.55	***0.001***	2.68	1.58-4.55	***0.001***

Le (Level of evidence); N (Number of Patients); PCR (Polymerase Chain Reaction); ihc (Immunohistochemistry); RDW (Red Cell Distribution Width); CDK (Cyclin-Dependent Kinase).

Bold letters mean statistical significance.

## Discussion

There is increasing recognition of the need for predictive biomarkers to assist in the management of chordomas. To the best of our knowledge, this is the first comprehensive review that summarizes prognostic biomarkers in chordoma that have been reported to date. The mainstay of treatment for localized chordomas is extensive resection with negative margins, but local recurrence and systemic metastases are common. Clinically reliable prognostic biomarkers that could aid in determining whether a particular chordoma is likely to recur would impact clinical decision-making, particularly with regard to the need for adjuvant therapy, the early incorporation of systemic therapy agents, and surveillance strategies.

PFS is a surrogate for OS (89). However, OS in diseases with more than 12 months of survival after disease progression/recurrence (like chordomas) is not a reliable endpoint, and a very large number of patients is required to power studies to detect statistically significant differences in solely in OS (90). Therefore, we focused on studies of biomarkers with a significant correlation for both endpoints. Because most of these studies are retrospective, potential confounders must be unmasked and multivariate analysis is an effective method of ranking various prognostic factors (91, 92). We found 13 biomarkers which are independently prognostic for both PFS and OS with statistical significance (**Table 1**). In each case, we compared the Hazard ratio (HR) and the Confidence Interval (CI). As a rule, the width of the CI correlates inversely with the precision of the biomarker. In other words, some biomarkers have a significant *p-value*, but the CI could be wide or includes the value of 1 (**Figures 2** and **3**). This situation decreases the precision of the biomarker when compared with others and is therefore an important consideration. We further discuss some of the 13 biomarkers below.

The expression of Programmed Death-1 (PD-1) receptor, its ligand, and the immune microenvironment in chordoma have been widely studied (30, 58). The PD-1/PD-L1 pathway is responsible for modulating T-cell activation (anti-tumor response) and exhaustion in cancer (93). Tumor cells express PD-L1 as an adaptive immune mechanism to escape this anti-tumor environment with a high number of CD8+ cells (Immunoscore) (58) or a high CD8+/Foxp3 ratio (30), consistently analyzed in our review. The blockade of this immune checkpoint signal has the potential clinical benefit of increasing the sensitivity of chordoma cells to T-cell mediated lysis. In this sense, PD-L1+ is currently a prognostic and therapeutic biomarker (10, 94). FDA-approved monoclonal antibodies currently used to block the PD-1/PD-L1 pathway include Pembrolizumab, Nivolumab, and Avelumab. Additionally, Nivolumab is currently being evaluated amongst rare CNS cancers including chordoma in 3 open clinical trials (NCT03173950, NCT02989636, NCT03623854). Even though the PD-1/PD-L1 pathway appears to be a very promising therapeutic and prognostic marker, the clones of anti-mouse PD-1 antibodies used are not consistent, and validation of them is needed to reach a wide reproducibility.

MicroRNA are small noncoding RNAs that play important roles in transcriptional gene regulation. Different miRNAs have been implicated in several cancers, including chordomas (30, 31, 42, 69, 88). Multiple proteins are involved in chordomagenesis, therefore it is unlikely that a single miRNA would provide optimal information regarding prognosis. Rather than focusing on a single miRNA, Huang et al. identified 6 prognostic miRNAs and developed a miRNA score (miRscore) based on integrated data. Using this approach, the miRscore was able to predict clinical outcome in chordoma (42). However, the main limitation of using this biomarker in clinical practice is the affordability along with ease and consistency of measurement using RT-PCR. An alternative approach is miRNA-seq but that is also hampered by technical complications limiting broad adoption (95).

Survivin is an apoptosis inhibitor protein widely expressed in tumors and non-terminally differentiated tissues (96). Ma et al. evaluated the expression of survivin in primary versus recurrent chordomas and demonstrated that survivin expression is elevated in recurrent tumors. Additionally, they found survivin levels were increased in recurrent tumors collected from the same patient, and expression of survivin was found to be an independent prognostic factor for progression (44). However, the lab technique used to quantify survivin expression was quantitative RT-PCR which is not practical assay from a clinical testing standpoint, although the use of survivin as a biomarker offers the advantage of measuring a single molecule as compared with the use of miRscore which measures 6 different miRNAs (42).

The Cyclin-dependent kinases (CDKs) are serine/threonine nuclear protein kinases involved in DNA transcription and cell cycle progression. In chordoma, CDK overexpression induces migration of tumor cells, proliferation, and tumor growth. In particular, the CDKN2A/p16INK4a/CDK4-6/RB pathway is a well-known cascade involved in tumor cell proliferation across multiple cancer types (97). The CDKN2A gene, and its protein product p16INK4a, have an inhibitory role over CDK4 and 6, decreasing cell proliferation. Mutations in CDKN2A gene have been studied as a prognostic biomarker in independent studies (38, 43, 50), but none have found a significant correlation with clinical outcomes. Only Sommer et al. (50) found that CDKN2A gene expression level correlated with a higher PFS *(p=.03)*; however, this correlation was not significant in multivariate analysis. CDK4 expression was found to be significantly associated with OS in a study by Yakkioui et al. (83), however, this was only in univariate analysis *(p=.021)*. These data suggest that this pathway has an unclear prognostic role in chordoma PFS or OS. Despite these uncertain results, the pathway is currently being evaluated for chordoma therapy. For example, palbociclib is an approved CDK4/6 inhibitor for chordomas with CDKN2A gene loss (98) and is currently being evaluated in a clinical trial (NCT03110744).

CDK12 overexpression increases the phosphorylation of antiapoptotic proteins, including Survivin and MCL-1 and correlates with shorter overall survival in breast cancer, ovarian cancer and gastric cancer (99, 100). Pichaya et al. measured the expression of CDK12 in tumor tissue in a cohort of 56 spine chordoma patients using immunohistochemistry, and grouped chordomas as those with low CDK-12 expression (less than 75% of the cells with positive nuclear staining) and those with high CDK-12 expression (more than 75% of the cells with positive nuclear staining) (52). The statistical analysis demonstrated a negative effect of this CDK in prognosis, however the results need to be interpreted with caution because the wide CI indicates lack of precision and little knowledge about the effect. While a study with a larger sample is required, the strength of this biomarker lies in the relatively easy assay technique, affordability and it resides upstream of other biomarkers implicated in poor clinical outcomes.

Somatic chromosome deletions have been studied as a type of genomic driver event in chordomas. Bai et al. conducted whole-genome sequencing of 80 skull base chordomas and evaluated the prognostic value of 17 somatic chromosomal events including gains of chromosomes 1q, 7p, and 7q, and deletions of 1p, 3, 4, 9, 10, 13q, 14q, 18, and 22q (43). Of these, the hemizygous deletion of 22q, where SMARCB1 gene is located, demonstrated a strong association with clinical outcomes. The homozygous deletion of SMARCB1 is a hallmark of poorly differentiated chordoma (101), but this finding suggests that even partial inactivation of SMARCB1 in conventional chordoma could be used as a prognostic biomarker. Nevertheless, the wide CI in this study highlights the need for validation with a larger sample. Although genome sequencing is an expensive and highly specialized assay technique, chromosomal deletion can be assessed with Fluorescence *in situ* hybridization (FISH) or spectral karyotyping (SKY) (102). In this regard, it is worth mentioning that Zenonos et al. present one of the few prospective studies with a strong statistical design (36). Using FISH, they found that skull base chordoma patients with a threshold percentage of cells with 1p36 and 9p21 deletions (more than 15% for 1p36 and more than 4% for 9p21) have a shorter PFS. Despite being a prospective study with thorough statistical design and promising HR, we did not include in **Table 1** because the study did not include OS and CIs in the analysis.

The last two biomarkers were assessed in blood samples and are the most promising in terms of affordability, limited invasiveness, reproducibility, assay technique, and required sample size (22, 46). Other studies have reported that increased levels of RDW or D-dimer are observed in many types of cancers and act as useful markers of adverse prognosis (103–106). In chordoma patients, a preoperative measurement above the cut-off values of 12.7 for RDW (in a spine chordoma population) and 840 μg/L for D-dimer (in a skull base chordoma population) were associated with a poor prognosis. However, these biomarkers do not provide an insight about the underlying disease mechanism, and they lack specificity (106–108).

After analyzing all of the available information gathered for this review, **Table 1** presents a set of biomarkers that have been demonstrated to correlate with both PFS and OS. While additional studies are needed to validate a narrower list of biomarkers with the strongest prognostic value, this proposed panel serves as a starting point. Given the techniques employed and the already available testing, there are biomarkers that can be readily incorporated into routine clinical setting. For example, preoperative D-dimer can be readily integrated as a potential prognostic biomarker due to its low-invasiveness, accessible measurement technique, and affordability. Also, the D-dimer study collected a significant cohort of patients (n=224) when compared with other biomarker studies and reported results using multivariate analysis with narrow CI (PFS, HR 2.68 [1.58, 4.55], p=.001; OS, 3.70 [1.81, 7.55], p=.001).

Furthermore, our inferential statistical analysis of the data has limitations because chordoma has intrinsic low incidence. Most of the studies report a limited patient cohort, making it difficult to assess the multivariate analysis of each biomarker. It is worth mentioning that none of the biomarkers met all the criteria mentioned in the introduction of this manuscript. They need to be validated in prospective studies with a larger sample size, and across multiple institutions. Affordability is one of the most important factors to consider for a biomarker that can be tested ubiquitously. The less expensive techniques used were serum protein analysis or hemocytometry, followed by immunohistochemistry and finally, some of the studies used expensive measurement techniques such as DNA sequencing (21, 43, 62), DNA microarrays (60, 86) and FISH (36, 45, 74). Also, when the lab technique is expensive or difficult to use, the studies are concentrated among the same centers, resulting in limited widespread integration. In order to use biomarker information to evaluate chordoma patients and enable clinical decision-making, high-quality prospective studies to evaluate the known potential biomarkers with strong biological underpinnings in chordomas are warranted. While retrospective studies remain useful in identifying potential new biomarkers, prospective trials with larger cohorts are essential for the eventual clinical translation of biomarkers.

## Conclusion

In this study we gathered all the prognostic biomarker studies reported to date according to PRISMA guidelines. More than a hundred biomarkers have been analyzed. The information is assorted, heterogenous, and lacks consistency in terms of markers used and tests performed. There is an increasing need to gather all these results to guide future validation studies to determine its clinical utility. A comprehensive understanding of this information can eventually help determine adjuvant treatments and surveillance strategies for patients with the worst prognosis. Further work is needed to validate a narrower list of biomarkers that correlate with overall survival, local recurrence, and metastatic spread with the strongest significance. Taken collectively, we encourage the comprehensive centers that evaluate and treat chordoma patients to collaboratively work to create a panel of prognostic biomarkers that can then be applied universally.

## Data availability statement

The original contributions presented in the study are included in the article/supplementary material. Further inquiries can be directed to the corresponding author.

## Author contributions

The authors confirm contribution to the paper as follows:

Study conception and design: FR and SR. Data collection: FR and SR. Analysis and interpretation of results: FR, CA-B, AC, AB, W-LW, AL, LR, FD, and SR. Draft manuscript preparation: FR, CA-B, KA, LR, FD, and SR. All authors reviewed the results and approved the final version of the manuscript.

## Acknowledgments

We thank Preeti Ramadoss, PhD for her editorial assistance in preparing this publication.

## Conflict of interest

The authors declare that the research was conducted in the absence of any commercial or financial relationships that could be construed as a potential conflict of interest.

## Publisher’s note

All claims expressed in this article are solely those of the authors and do not necessarily represent those of their affiliated organizations, or those of the publisher, the editors and the reviewers. Any product that may be evaluated in this article, or claim that may be made by its manufacturer, is not guaranteed or endorsed by the publisher.

## Abbreviations

PRISMA, Preferred Reporting Items for Systematic Reviews and Meta-Analyses; FDA ,Food and Drug Administrations; PFS, Progression Free Survival; OS, Overall Survival; PD-1, programmed cell death protein 1; CDK, cyclin dependent kinases.
